# Post-Surgical Evaluation in Stress Urinary Incontinence by Transperineal Ultrasound and Quality of Life Questionnaire—A Retrospective Study

**DOI:** 10.3390/diagnostics16172722

**Published:** 2026-08-26

**Authors:** Melinda Ildiko Mitranovici, Laura Georgiana Caravia, Ion Petre, Septimiu Voidazan, Daniela Ceana, Raluca Moraru, Liviu Moraru

**Affiliations:** 1 Department of Obstetrics and Gynecology, Emergency County Hospital Hunedoara, 14 Victoriei Street, 331057 Hunedoara, Romania; mitranovicimelinda@yahoo.ro; 2 Division of Cellular and Molecular Biology and Histology, Department of Morphological Sciences, “Carol Davila” University of Medicine and Pharmacy, 050474 Bucharest, Romania; 3 Department of Functional Sciences, Medical Informatics and Biostatistics Discipline, “Victor Babes” University of Medicine and Pharmacy, 300041 Timisoara, Romania; petre.ion@umft.ro; 4 Department of Epidemiology, “George Emil Palade” University of Medicine, Pharmacy, Sciences and Technology, 540142 Targu Mures, Romania; septimiu.voidazan@umfst.ro; 5 Department of Public Health and Management, “George Emil Palade” University of Medicine, Pharmacy, Sciences and Technology, 540142 Targu Mures, Romania; daniela.ceana@umfst.ro; 6 Department of Anatomy, “George Emil Palade” University of Medicine, Pharmacy, Sciences and Technology, 540142 Targu Mures, Romania; raluca.moraru@umfst.ro (R.M.); liviu.moraru@umfst.ro (L.M.)

**Keywords:** stress urinary incontinence, transperineal ultrasound, quality-of-life questionnaire, diagnosis

## Abstract

Urinary incontinence, the involuntary leakage of urine, significantly impacts middle-aged women. **Background/Objective**: In response to the failure of traditional diagnostic methods, transperineal ultrasound (TPUS) imaging was developed. The aim of our study was to identify the utility of TPUS in combination with a quality-of-life questionnaire in stress urinary incontinence evaluation and its reflection in clinical outcomes. **Methods:** This retrospective study was conducted between 18 March 2022 and 31 December 2024 in the County Hospital Hunedoara, Romania, Department of Obstetrics and Gynecology. It included 58 patients who underwent surgery for stress urinary incontinence and a control group consisting of 50 patients without SUI. Quality of life scores were recorded preoperatively and 6 months postoperatively, and ultrasound examination was performed. Four variables (Q-type test, the expansion of the funnel shape of the proximal urethra, Doppler assessment, and bladder neck descent) were measured, and their preoperative and postoperative values were compared in the study and control groups. **Results:** Significant statistical differences were observed in the variables measured through TPUS before and after surgery in the study group (*p* = 0.0001). These characteristics were significantly different in the study group compared with control group (*p* = 0.0001). All women in the study group reported a severe impact on their quality of life compared with the control women (10% reported a moderate level) (*p* = 0.000). Additionally, quality of life was significantly improved in the study group after surgery (*p* = 0.0001). **Conclusions**: Transperineal ultrasound is a noninvasive, cost-effective, and highly reliable imaging technique used to assess SUI. Questionnaires are also useful in evaluating patients’ quality of life and satisfaction after surgery.

## 1. Introduction

Urinary incontinence is the involuntary leakage of urine. The weakening of the pelvic floor muscles leads to this disease, which is a significant global health problem impacting middle-aged women in particular [1,2]. Because stress urinary incontinence (SUI) is a prevalent condition with a high impact on quality of life, transperineal ultrasound (TPUS) imaging was developed to diagnose SUI. Urethral mobility is a key factor in the development of SUI, and TPUS can provide a clear visualization of this movement; however, there are little data in the literature to validate its standardization [1]. Using TPUS imaging, we can diagnose stress urinary incontinence (SUI) by comparing the urethral angle (α), posterior ureterovesical angle (β), and bladder neck descent (BND) during rest and the Valsalva maneuver [3]. The retrograde vesical urine flow helps illustrate vesical neck incompetence, dynamic funneling, and the loss of functional urethral closure pressure during increased abdominal stress [3]. Further research is needed to standardize these measurements and validate their clinical utility; importantly, there is also little information on normal values [4,5].

Determining the optimal placement of periurethral material requires determining the urethral length for SUI treatment. Urethral length can be measured with a catheter and compared with the TPUS measurement [6]. Bladder neck descent and the results of the urodynamic test of maximal urethral pressure are the only independent predictors of SUI [7].

As traditional diagnostic tools have failed in diagnosing SUI, specific questionnaires and other diagnostic tools have been developed [2]. Questionnaires have been identified as valuable tools for identifying and diagnosing SUI, as they can be used to evaluate its impact on quality of life and measure satisfaction following treatment [8,9].

However, controversy about the correct choice of diagnostic and treatment methods persists. A personalized approach to diagnosis is required [2]. There is a need to establish more standardized and objective parameters for the diagnosis of urinary incontinence. More accurate analyses and comparisons of the parameters will support a more clinically useful diagnostic test, ensuring reliable and reproducible results [4].

The aim of our study was to determine the utility of transperineal ultrasonography (TPUS) in diagnosing stress urinary incontinence and its reflection in clinical outcomes.

## 2. Materials and Methods

The present retrospective study based on prospectively collected data was conducted between 18 March 2022 and 31 December 2024 in the County Hospital Hunedoara, Romania, Department of Obstetrics and Gynecology. It included 58 patients who underwent trans-obturator tension-free surgery or other surgery for stress urinary incontinence, such as TOT and vaginal hysterectomy, and a control group consisting of 50 patients without SUI. Female adult patients with symptomatic SUI satisfied the inclusion criteria. Patients with previous anti-incontinence surgery, other types of urinary incontinence, patients who used other therapies for symptoms of incontinence, and patients with pathologies such as diabetes and neurological diseases that generate incontinence were excluded. Also, patients who lost follow-up were excluded. All participants provided written informed consent, and our research was conducted in accordance with the Declaration of Helsinki with the approval of the Ethics Committee of County Hospital Hunedoara, Romania (No. 3511/18.02.2025).

Quality-of-life scores were recorded preoperatively and 6 months postoperatively. A standardized questionnaire was used to assess SUI. A positive cough test on physical examination and urinary leakage were observed and recorded. Symptoms, especially during daily activities, as well as emotional well-being and quality of life, were assessed using the Pelvic Floor Impact Questionnaire (PFIQ-7). A 4-point Likert scale was used, with 1 = “not at all”, 2 = “somewhat”, 3 = “moderately”, and 4 = “quite a bit” [10]. The average score of answered items for each subscale (range 0 to 3) was computed. Each subscale average was multiplied by 100/3 (or 33.3) to scale it from 0 to 100. The PFIQ-7 scores can be categorized into low (<100), moderate (101–200), and severe (201–300), where a lower score indicates a positive effect from treatment and improved quality of life.

Ultrasound examination was performed using a Voluson S10 Expert sonography machine (GE Healthcare, Milwaukee, WI, USA). A trans-perineal ultrasound was performed to assess the pre- and postoperative urethral length, urethral mobility, bladder neck descent, and structural changes during rest and the Valsalva maneuver (straining). The evaluation was made by the same sonographer each time, who was trained in this type of sonography. The examiner was blinded to continence status or surgical time point. The ultrasound testing was performed in the standard position for gynecologic examination, with women lying on their back with their legs bent at the knees. A full bladder (120–400 mL) was required. The transperineal US is performed at the level of the vaginal vestibule. After identifying the inferior edge of the symphysis pubis in the sagittal axis, the urethra, bladder and urethra–vesical junction are exposed.

Urethral length, measured using a catheter before and after surgery, was compared with the transperineal ultrasound measurements. The distance between the inflated balloon of the Foley catheter and the urethral meatus was considered and then compared with the transperineal ultrasound measurements for the posteroinferior margin of the pubic symphysis to the bladder neck for accuracy [2,6].

We measured bladder neck descent (BND), with a BND > 13.3 mm considered increased. In resting position: we measured with the patient supine and having a partially full bladder, locating the bladder neck position relative to the chosen reference line. Then, we took maximum strain measurements during a prolonged Valsalva maneuver, tracking the downward and posterior displacement of the bladder neck. Standardizing the Valsalva maneuver replaces subjective patient strain with a goal-directed protocol using a pressure device, targeting 40 mm Hg of intraoral pressure held for 10 to 15 s [10].

In patients with stress urinary incontinence (SUI), the Q-tip test measures urethral mobility and descent during straining (like coughing or performing a Valsalva maneuver), and its normal value is <30 of upward deflection from the horizontal plane at rest. A positive Q-tip test of >30 indicates weakened pelvic floor support (urethral hypermobility) and the descent of the bladder neck. Furthermore, bladder neck funneling, in which the proximal urethra opens into a conical shape during the Valsalva maneuver, is characteristic of SUI, with a diameter > 6 mm strongly associated with SUI [6,11]. The reverse flow of urine from the urethra to the bladder refers to urine being pushed backward toward the bladder during coughing and is correlated with decreased vascularity of the tissue surrounding the urethra in women with SUI [3,11]. Such measurement is not recognized as a standard diagnostic parameter for stress urinary incontinence (SUI); standard ultrasound assessments for SUI focus on evaluating urethral hypermobility via bladder neck descent. Standard ultrasound machine settings and high inter-observer reproducibility govern dynamic transperineal/pelvic floor Doppler assessments for stress urinary incontinence (SUI), though the literature focuses primarily on anatomical mobility (bladder neck descent and urethral rotation) rather than specific “reverse flow” Doppler jets. Low-velocity scale settings (typically set to minimal low pulse repetition frequency, ~2–5 cm/s) are used to capture low-velocity fluid shifts or fluid escape through the urethral meatus during provocation. Optimized high color gain is used without introducing excessive artifact noise, with low wall filters to prevent the obscuring of minor shifting fluid signals during a Valsalva maneuver or cough stress test. The dynamic range and focus zone are placed precisely at the level of the bladder neck and proximal urethra, with a narrow sector width to maximize frame rates (temporal resolution) [12,13].

Surgical procedures for SUI were performed under spinal anesthesia. For the trans-obturator tape (TOT), we used the synthetic single-thread polypropylene tape SOFT LIFT^®^ with an associated tunneling device (FANCOFIL, FANCOFEN) (COUSIN BIOTECH 8, rue de l’Abbé Bonpain 59117, Wervicq-Sud, France). A total of 30 patients required complex interventions for genital prolapse, in 9 cases associated with uterine fibroids. For these cases, in addition to TOT, we performed a vaginal hysterectomy. The procedures were all done by the same team. Tape tensioning was achieved using an intraoperative cough test. The duration of hospitalization was between 3 and 5 days. No intra- or postoperative complications were reported based on the Clavien–Dindo Classification [14].

Follow-up visits were scheduled for 6 months after surgery and included a cough test, quality of life questionnaire, and transperineal ultrasound examination. The follow-up visits were conducted by the surgeons who performed the surgery, then evaluated by the first author. The objective cure rate was defined as no leakage during the cough test with a bladder filling of approximately 300 mL.

The data were analyzed using descriptive statistical methods.

Statistical analysis was performed using statistical software: SPSS IBM V.23 for Windows and Microsoft Excel 365. Quantitative variables (age, BMI) were compared using *t* test. The Chi-square/Fisher test was used for categorical variables to determine associations or comparisons. For pre- and postoperative comparisons, the McNemar test was applied. Continuous parameters are expressed as the mean ± standard deviation (SD), and categorical variables are presented as the number and percentage. The chosen materiality threshold was alpha = 0.05, and *p* was considered significant when *p* ≤ alpha.

## 3. Results

The characteristics of the study patients and controls are compared in Table 1. All the demographic and clinical variables included were relevant to SUI development, with significant differences observed between the study group and controls. Most of these factors—including age, BMI, parity, vaginal deliveries, menopausal status, and diabetes—are well-established prognostic factors for stress urinary incontinence (SUI).

For the four variables (Q-type > 30 degrees; the expansion of the funnel shape of the proximal urethra (sphincter insufficiency); the Doppler assessment of reversal of urine flow from the urethra to the bladder; rest stress distance > 13.3mm), we applied the McNemar test to compare the study groups’ preoperative and postoperative measurements.

For the variable Q-type angle > 30 degrees, there were 58 preoperative patients and only 1 postoperative patient (significant statistical differences, *p* value 0.0001, Table 2).

For the Doppler variable—reverse flow of urine from the urethra to the bladder—31 preoperative presence (yes) was observed for 31 participants, while only 1 had postoperative reverse flow. Twenty-seven patients had neither (significant statistical differences, *p* value 0.0001, Table 3).

For the funnel-shaped expansion of the proximal urethra (sphincter incompetence), of the 52 patients who had a preoperative presence, only five had a postoperative presence (significant statistical differences, *p* value 0.0001, Table 4).

For a bladder neck descent > 13.3 mm, of 57 patients who had a preoperative presence, only seven had a postoperative presence (significant statistical differences, *p* value 0.0001, Table 5).

All characteristics were compared between the study group before surgery and the control group to highlight TPUS’s efficacy as a diagnostic tool. All variables were significantly modified (Table 6).

**Figure 1:**
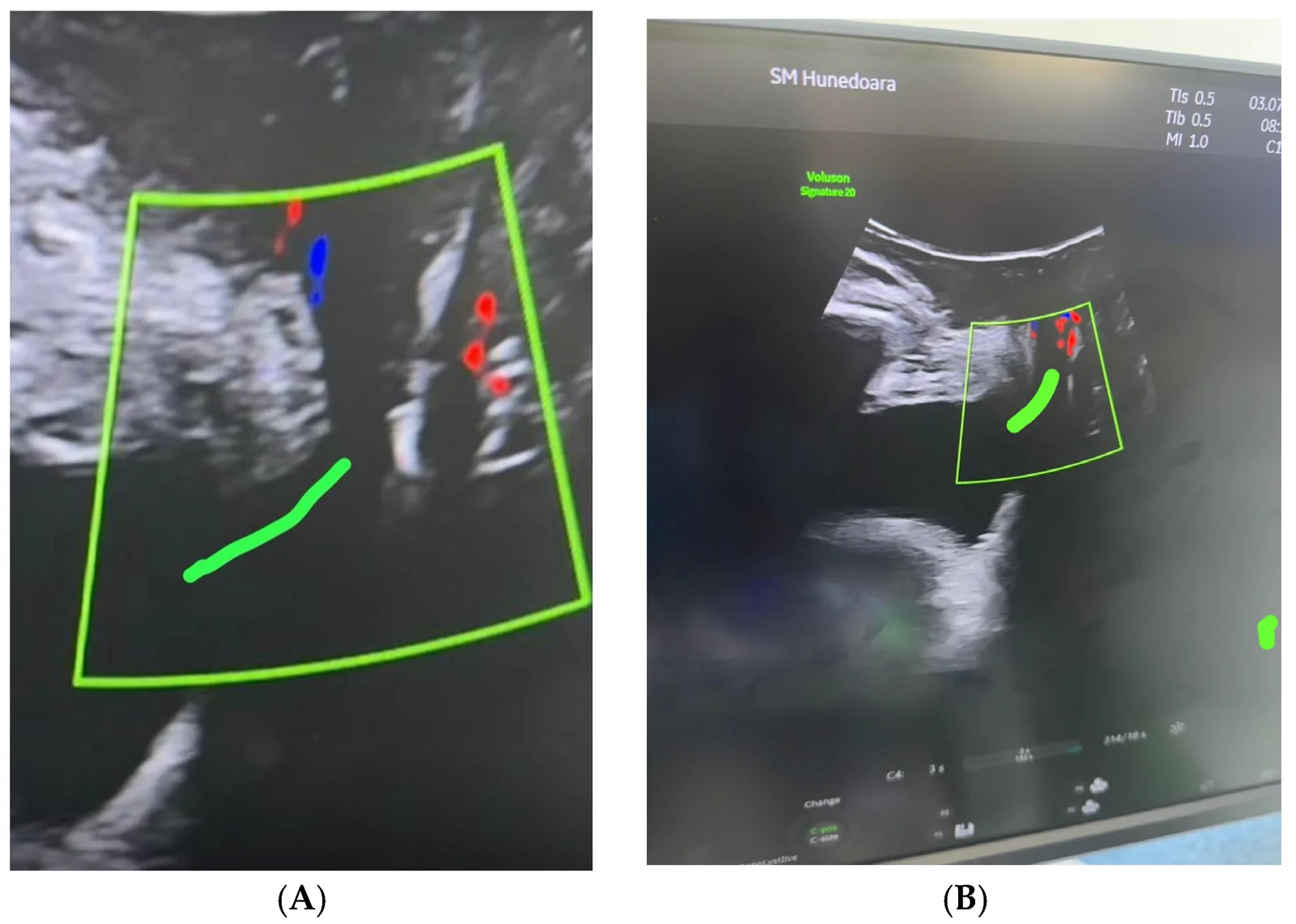

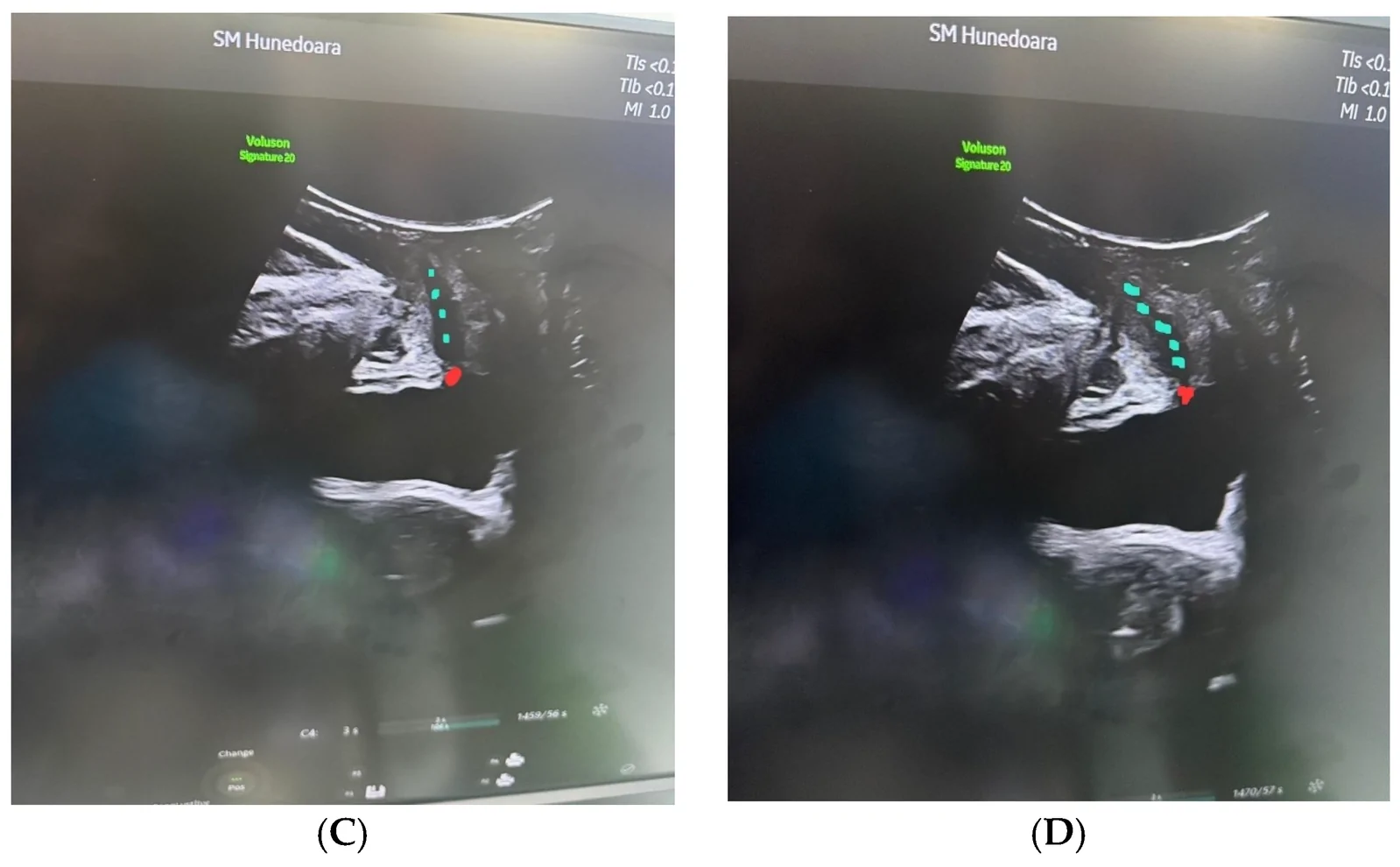
TPUS images of SUI cases: (**A**,**B**) the funel shape of urethra during the Valsalva maneuver in midsagital plane; (**C**) the urethra (green) and bladder neck (red) at rest; (**D**) urethral mobility and bladder neck descent during Valsalva manoever in a SUI case. (Red and blue is the Doppler signal in the green frame).

Some examples of TPUS images in the case of SUI are presented below (Figure 1).

As a diagnostic test, the cough test to determine urinary incontinence revealed urine leakage in all patients compared with women without incontinence. It also contributed to the postoperative evaluation, showing an improvement from 58 women with a positive cough test to just two after surgical intervention.

The quality-of-life questionnaire helped evaluate patients with SUI compared with women without incontinence. It was also useful in the postoperative evaluation of women with SUI. All women with SUI reported a severe impact on quality of life, while in the control group, 45 (90%) out of 50 women had good quality of life, and five (10%) reported a moderate impact. The difference is statistically significant at *p* = 0.0001 (Table 7).

While all patients with SUI reported a severe impact on quality of life before surgery, after surgery, we found an improvement in quality of life in all patients; in some cases, improvement increased from severe to moderate for those with complex surgery such as additional hysterectomy (17.25%), and in others it improved from severe to good (82.75%). Significant differences were observed (*p* < 0.0001).

**Figure 2:**
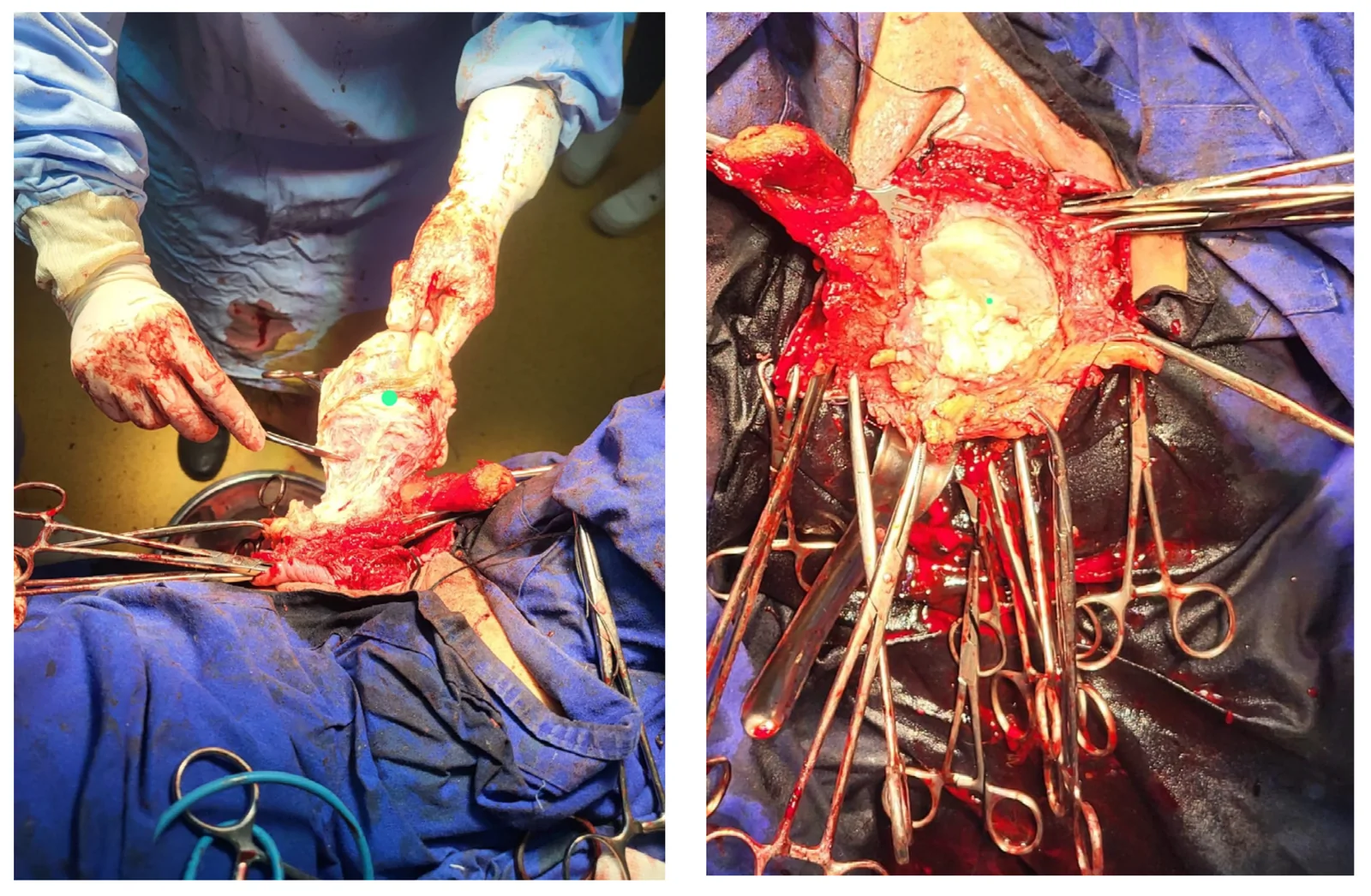
Uterine prolapse and SUI: surgical intervention. The green color marks the uterus with a bulky fibroid.

The most important improvement was observed in women with complex SUI associated with genital prolapse. In the figure below, we present a prolapse associated with uterine myoma and SUI (Figure 2).

## 4. Discussion

Stress urinary incontinence is a significant global health issue with a considerable impact on quality of life for middle-aged women. Transperineal ultrasound and specific questionnaires have become useful diagnostic tools for pelvic floor disorders [2]. Several studies in the literature highlight the utility of this approach in the assessment of SUI. Quality-of-life questionnaires and advancements in imaging techniques have improved the management of pelvic floor function in a reproducible and valid way [2].

In our study, all of the patient characteristics reported significantly influenced the development of stress urinary incontinence: postmenopausal status, vaginal delivery, estrogen therapy, age, BMI, diabetes mellitus, parity, fibroma, and genital prolapse. These features are well established risk factors for SUI [15,16].

Significant pre- and postoperative differences were observed for the four variables measured with transperineal ultrasound (Q-type angle > 30 degrees; the expansion of the funnel shape of the proximal urethra (sphincter insufficiency); the Doppler assessment of reversal of urine flow from the urethra to the bladder; rest stress distance > 13.3 mm) in the study group (*p* < 0.0001). From this perspective, these variables, measured through TPUS, have an important role in follow-up after surgery and in quantifying surgical technique efficacy. These characteristics were also measured in the control group, with a statistically significant difference compared with the study group, showing their diagnostic value in SUI (*p* < 0.0001).

The Q-tip/straightening test was selected because a 30-degree deviation is widely treated as a critical clinical tipping point that changes the surgical plan. It serves as a practical, reproducible benchmark in evaluating post-degloving or post-repair outcomes, helping to reduce reliance on purely subjective visual estimates. An upward or downward movement exceeding 30 degrees from the horizontal plane is standard for identifying abnormal laxity of the supporting tissues [17].

According to our findings, the cough test is a relevant diagnostic test for women with SUI: urine leakage was demonstrated in all patients compared with women without incontinence. The quality-of-life questionnaire was used successfully in diagnosing SUI; all women in the study group reported a severe impact on their quality of life compared to the normal women (10% reported a moderate level) (*p* < 0.000). After surgery, quality of life improved significantly in the study group (*p* < 0.0001).

Chen et al. reviewed 13 studies; out of 1563 participants, no significant differences were observed in age or parity between SUI patients and women without incontinence, and BMI was only slightly higher in the SUI group than in the normal population. Significant differences were observed in the α angle, β angle at rest, and during the Valsalva maneuver in the bladder neck descent. Their research revealed a potential use of transperineal ultrasound in diagnosing SUI [18]. In our study group, the BND was significantly higher than in controls (*p* < 0.0001); parity, age, and BMI were also found to be relevant in SUI development.

Transperineal ultrasound was evaluated as diagnostic tool in Turkouglu et al.’s study on 50 women with SUI and 50 continent women. The Q-tip test was performed, and the α and β angles were evaluated. A significantly higher value was obtained in women with SUI during the Valsalva maneuver (*p* < 0.001) compared to measurements at rest. The Q-tip test and angle and α value were strongly correlated (*p* = 0.000; r = 0.890). The bladder neck descent was significantly higher in the SUI group (*p* < 0.001). In this regard, transperineal ultrasound is a practical, non-invasive method for the evaluation of SUI [3]. Our research results support using TPUS as a diagnostic tool, with the bladder neck descent and Q-type test results being strongly correlated with SUI (*p* < 0.0001).

Alper et al. used transperineal and transvaginal ultrasonography and compared the posterior ureterovesical angles, measured by ultrasonography, of 50 women with SUI (group I) and 50 control cases (group C). According to their study, ultrasonography is not a reliable, useful diagnostic tool in the evaluation of SUI, especially transvaginal ultrasounds [19]. In contrast, Yıldırım et al., in a retrospective study on 40 patients who underwent TVT surgery, reported that TPUS is a useful and objective method of monitoring clinical outcomes in patients undergoing transvaginal tape (TVT) surgery for stress urinary incontinence (SUI) [20].

TPUS can be also considered a valuable tool to evaluate failed trans-obturator mid-urethral synthetic sling MUS intervention [21,22].

Even if, according to our research, TPUS associated with specific questionnaires is a highly relevant diagnostic tool in SUI, ultrasound measurements cannot replace urodynamic testing according to Dietz et al. [7]. Questionnaires can be applied to evaluate SUI before and after different surgical techniques; trans-perineal ultrasounds can be used not only to diagnose the condition but also to evaluate outcomes at six months postoperative with high efficacy [23,24]. In our research, quality-of-life questionnaires revealed a significant difference between the study group and controls and were also relevant in evaluating postoperative outcomes in patients with SUI. A significant improvement in quality of life was observed in all SUI patients, improving in some from severe to moderate (17.25%) and in others from severe to good (82.75%) (*p* < 0.0001).

Different researchers evaluated different ultrasonographic variables and considered them highly relevant for SUI diagnosis. The funnel-shaped expansion of the proximal urethra was reported by Lugo et al. to be be highly relevant for intrinsic sphincter deficiency, which is a major cause of SUI [25]. In contrast, Bergstrom stated that urethral sphincter failure is not the predominant cause of female stress urinary incontinence [26]. In our research, funnel-shaped expansion was strongly correlated with SUI.

To observe vascularization in periurethral tissue, we used color Doppler, which proved valuable in evaluating SUI. We obtained a positive correlation of reverse urine flow observed with Doppler ultrasonography and SUI (*p* < 0.0001). According to Mascarretti, the Doppler ultrasound is an important instrument that can help diagnose SUI [27]. In Doppler evaluations of the urethra, di Bella also found statistically significant differences between incontinent women affected by the postmenopausal period [28]. In contrast, Lone observed no changes in vascularity parameters in women with SUI after surgery [29].

Transperineal ultrasound (TPUS) is a non-invasive, dynamic, and highly reliable imaging technique for the assessment of stress urinary incontinence (SUI). In many cases, it has superior diagnostic utility over traditional methods such as the Foley catheter and Q-tip tests, both of which were used in this study. Studies show high agreement between TPUS (3D) and Foley catheter techniques for measuring urethral length. TPUS offers the real-time visualization of pelvic floor structures, including the bladder neck, urethra, and pubic symphysis, allowing for dynamic assessment during the Valsalva maneuver (straining) that a static catheter cannot provide [3,6,11]. However, standardization in quantifying urethral movement is required to provide more information and reduce inter-observer differences in measurements. This can be achieved using the symphysis pubis as a reference coordinate system to probe urethral movements during Valsalva maneuvers [1,22]. Transperineal ultrasound provides reproducible images of pelvic floor conditions, which are required in the assessment of urogynecological conditions [22].

TPUS has some advantages; for example, its low cost, lack of radiation, comfort for the patient compared with traditional diagnostic tools, dynamic evaluation, and the ability to detect occult SUI. However, it has some limitations. For example, it requires training, lacks standardization across all centers, and there is a need for larger studies to confirm thresholds.

Efforts are ongoing to standardize TPUS reporting, including developing automated, computer-vision-based, real-time quantification methods for urethral mobility to eliminate inter-observer variability. Future study designs have been developed for TPUS, such as machine-based deep-learning on TPUS static images with high value in clinical practice [30,31].

A strength of our study is that we used several TPUS measurements as variables and diagnostic tools to evaluate SUI and to quantify the postoperative situation in combination with a quality-of-life questionnaire. A limitation of our study is that it is a retrospective single-center study. The sample size was constrained by available retrospective data. Furthermore, the questionnaire did not aid in risk stratification. However, our results align with those from the literature, and our study combined the questionnaires with TPUS, which helped us understand to what extent surgical procedures improve various aspects of quality of life with high clinical relevance.

## 5. Conclusions

Transperineal ultrasound (TPUS) is a noninvasive, cost-effective, and highly reliable imaging technique used to assess SUI. It provides the real-time visualization of pelvic structures, particularly during the Valsalva maneuver, to measure bladder neck mobility and mobility-related dysfunction. Key metrics, such as bladder neck descent (BND) and urethral rotation, help diagnose SUI and evaluate postoperative outcomes. In addition, questionnaires such as the PFIQ 7 are relevant to evaluating patients’ quality of life. However, these are exploratory findings from a small, single-centre, retrospective cohort.

## Figures and Tables

**Table 1:** Characteristics of patients in the two groups.

Variables		Groups		*p* Value
		Control (No. = 50)	Study (No. = 58)	
Postmenopausal status	Yes	39 (78.0)	56 (96.6)	0.003
Parity	1	32 (64.0)	1 (1.7)	0.0001
	2	18 (36.0)	16 (27.6)	
	3	0 (0.0)	22 (37.9)	
	4 or more	0 (0.0)	19 (32.7)	
Vaginal deliveries	Yes	29 (58.0)	58 (100.0)	0.003
Estrogen therapy	Yes	6 (12.0)	40 (69.0)	0.001
Fibroma	Yes	0 (0.0)	9 (15.5)	0.004
Prolapse	Yes	0 (0.0)	30 (51.7)	0.0001
Diabetes	Yes	1 (2.0)	7 (12.1)	0.046
Age	Mean ± SD	57.34 ± 6.89	66.14 ± 8.85	0.0001
BMI	Mean ± SD	24.88 ± 1.25	27.72 ± 1.70	0.0001
Preoperative	Good	45 (90.0)	0 (0.0)	0.0001
	Moderate	5 (10.0)	0 (0.0)	
	Severe	0 (0.0)	58 (100.0)	

Note: Data are presented as number and percentage: Chi-square/Fisher test was applied; Data are presented as mean ± standard deviation: *t* test was applied.

**Table 2:** McNemar test for Q-tip angle > 30 degrees.

*p* < 0.0001	Q-tip > 30 Degrees (pre-op)		
Q-tip angle > 30 degrees (postop)	No	Yes	
No	0	57	57 (98.3%)
Yes	0	1	1 (1.7%)
	0	58	58 (100.0%)

**Table 3:** McNemar test for Doppler assessment of reverse flow of urine from the urethra to the bladder.

*p* < 0.0001	Doppler: Reverse Flow of Urine from the Urethra to the Bladder (pre-op)		
Doppler: Reverse flow of urine from the urethra to the bladder (postop)	No	Yes	
No	27	30	57 (98.3%)
Yes	0	1	1 (1.7%)
	27	31	58 (100.0%)

**Table 4:** McNemar test for funnel-shaped expansion of proximal urethra (sphincter incompetence).

*p* < 0.0001	Funnel-Shaped Expansion of Proximal Urethra (Sphincter Incompetence) (pre-op)		
Funnel-shaped expansion of proximal urethra (sphincter incompetence) (post-op)	No	Yes	
No	6	47	53 (91.4%)
Yes	0	5	5 (8.6%)
	6	52	58 (100.0%)

**Table 5:** McNemar test for rest stress distance > 13.3 mm.

*p* < 0.0001	Bladder Neck Descent > 13.3 mm (pre-op)		
Rest stress distance > 13.3 mm (post-op)	No	Yes	
No	1	50	51 (87.9%)
Yes	0	7	7 (12.1%)
	1	57	58 (100.0%)

**Table 6:** TPUS variables for diagnosis (preoperative): study group vs. controls.

Variables	Groups		Total (No. = 108)	*p* Value
	Control (No. = 50)	Study (No. = 58)		
Q-tip angle > 30 degrees (yes)	0 (0.0)	58 (100.0)	58 (53.7)	0.0001
Funnel-shaped expansion of proximal urethra (sphincter incompetence) (yes)	1 (2.0)	52 (89.7)	53 (49.1)	0.0001
Doppler: reverse flow of urine from the urethra to the bladder (yes)	1 (2.0)	31 (53.4)	32 (29.6)	0.0001
Bladder neck descent > 13.3 mm (yes)	2 (4.0)	57 (98.3)	59 (54.6)	0.0001

Note: Data are presented as number and percentage: Chi-square/Fisher test was applied.

**Table 7:** Quality-of-life questionnaire: study groups vs. controls.

*p* < 0.0001		Groups		Total
		Control (No. = 50)	Study (No. = 58)	
Pre-op	Good	45 (90.0)	0 (0.0)	45 (41.7)
	Moderate	5 (10.0)	0 (0.0)	5 (4.6)
	Severe	0 (0.0)	58 (100.0)	58 (53.7)
Total		50 (46.3)	58 (53.7)	108 (100.0)

Note: Data are presented as number and percentage: Chi-square/Fisher test was applied.

## Data Availability

The original contributions presented in this study are included in the article. Further inquiries can be directed to the corresponding author.

## Abbreviations

The following abbreviations are used in this manuscript:

SUI	Stress urinary incontinence
TPUS	Transperineal ultrasound
BND	Bladder neck descent
PIFQ-7	Pelvic Floor Impact Questionnaire
